# Fire needle acupuncture at Neiyingxiang (EX-HN 9) in the treatment of moderate and severe refractory perennial allergic rhinitis: A case report

**DOI:** 10.1097/MD.0000000000037060

**Published:** 2024-01-26

**Authors:** Jianwei Ai, Suying Guo, Yaqin Wang, Yuezhi Kang, Man Wang, Jingyi Zhao, Shaoting Huang, Junge Wang

**Affiliations:** a Department of Otorhinolaryngology Head and Neck Surgery, Beijing Hospital of Traditional Chinese Medicine, Capital Medical University, Beijing, China.

**Keywords:** acupuncture, case report, fire needle, Neiyingxiang, perennial allergic rhinitis

## Abstract

**Rationale:**

In recent decades, the incidence of perennial allergic rhinitis (PAR) has been increasing annually. However, some patients could not achieve adequate symptomatic relief with routine pharmacological treatment. Consequently, there exists an urgent clinical imperative for the development of safe and efficacious treatments with sustained therapeutic impact to ameliorate the symptomatic burden and enhance the quality of life.

**Patient concerns:**

The patient was a 35-year-old woman. She had suffered moderate and severe refractory PAR for decades and failed to sustain symptom mitigation from regular treatment.

**Diagnoses:**

Perennial allergic rhinitis.

**Interventions:**

The patient underwent a 4-week course of fire needle acupuncture at Neiyingxiang, administered weekly, during which all allopathic medication was discontinued.

**Outcomes:**

The total nasal symptoms score, total non nasal symptoms score, rhinoconjunctivitis quality of life questionnaire, and the total nasal resistance of the patient were decreased after treatment and achieved symptomatic relief. Follow-up conducted 3 months post-treatment revealed enduring symptom relief, with only sporadic nasal congestion elicited by cold stimulus.

**Lessons:**

This case proves that, fire needle acupuncture at Neiyingxiang may be beneficial in treating moderate and severe refractory PAR patient and have a lasting effect.

## 1. Introduction

Allergic rhinitis constitutes a pervasive inflammatory pathology affecting the nasal mucosa, characterized by symptoms including nasal pruritus, sneezing, rhinorrhea, and nasal obstruction. In recent years, the global prevalence of this condition has witnessed a marked escalation, thereby impacting approximately 400 million individuals and engendering significant socio-economic ramifications as well as substantially deteriorating the quality of life of affected individuals.^[1]^ The manifestation of symptoms for a duration extending beyond 4 days per week for a consecutive 4-week period categorizes the condition as perennial allergic rhinitis (PAR). Furthermore, when such manifestations impinge upon sleep quality, occupational productivity, or routine daily activities, the condition is classified as moderate-to-severe allergic rhinitis.^[2]^ For such patients, chronic administration of either oral pharmacotherapeutics or nasal corticosteroid sprays is often requisite for symptomatic management. However, the therapeutic efficacy of such interventions is predominantly transient, and symptom recurrence is commonplace upon cessation of medication. Approximately one-third of patients do not achieve adequate symptomatic relief with pharmacological treatment alone.^[3]^ Consequently, there exists an urgent clinical imperative for the development of safe and efficacious treatments with sustained therapeutic impact to ameliorate the symptomatic burden and enhance the quality of life.

The fire needle acupuncture, an ancient modality in traditional Chinese medicine, employs a rapidly inserted heated needle for therapeutic applications. Compared to conventional acupuncture, the fire needle method augments physiological responsiveness via thermal stimulation.^[4]^ While previous studies have primarily focused on cutaneous acupoints for fire needle intervention, often resulting in local cutaneous injury and consequent patient aversion.^[5]^ No extant literature has explored the utilization of intranasal acupoint fire needle technique in the treatment of allergic rhinitis. In the present study, we implemented fire needle acupuncture at the Neiyingxiang (EX-HN 9) acupoint within the nasal mucosa and observed durable therapeutic outcomes.

## 2. Case report

### 2.1. Clinical presentation

A 35-year-old female patient presented to the Otolaryngology clinic at the Beijing Hospital of Traditional Chinese Medicine on October 28, 2022, with a decadal history of recalcitrant allergic rhinitis symptoms, including nasal congestion, rhinorrhea, nasal pruritus, and sneezing. These symptoms were exacerbated by fluctuations in thermal environments and imposed a considerable detriment to her quality of life. Prior therapeutic interventions comprised antihistamines and corticosteroid nasal sprays. Initial symptomatic control was achieved during the first 3 years of pharmacological intervention. However, subsequent treatment failed to sustain symptom mitigation. She had tried allergen-specific sublingual immunotherapy, but failed to relieve all the symptoms. Recent exacerbation coincided with the onset of colder climatic conditions. A comprehensive diagnostic evaluation, including nasal endoscopy and sinus computed tomography, revealed bilateral inferior turbinate hypertrophy and edema, with resultant narrowing of the nasal passages but excluded chronic sinusitis and nasal polyps. Allergen testing implicated mite and mold sensitivities. Concomitant symptoms included cold intolerance, excessive perspiration, and general malaise, as well as suboptimal sleep quality. Nasal examination showed pale nasal mucosa with bilateral inferior turbinates swollen and edema, which could not see the middle turbinates. Accordingly, a diagnosis of moderate-to-severe PAR was rendered.

### 2.2. Clinical intervention

**Figure 1.**
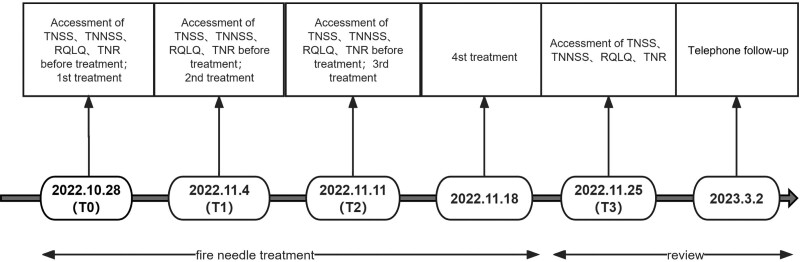
The timeline of fire needle acupuncture at EX-HN 9 for the PAR patient. EX-HN 9 = Neiyingxiang, PAR = perennial allergic rhinitis.

The patient underwent a 4-week course of fire needle acupuncture, administered weekly, during which all allopathic medication was discontinued. Symptomatic severity was assessed via the total nasal symptoms score, total non nasal symptoms score, rhinoconjunctivitis quality of life questionnaire questionnaires and total nasal resistance (TNR) at the commencement, midpoint, and conclusion of the treatment regimen (Fig. 1). The bilateral TNR was measured at a nasal pressure of 150 Pa. Subsequent telephonic follow-up was undertaken to gauge the sustainability of treatment outcomes. Symptomatic relief was achieved after the initial treatment session, with substantial reduction in nasal congestion and rhinorrhea. Following the second session, the patient reported near-total amelioration of allergic rhinitis symptoms and expressed high levels of treatment satisfaction. Two additional sessions were conducted to consolidate therapeutic gains, during which no symptomatic recurrence was observed. Telephonic follow-up conducted 3 months post-treatment revealed enduring symptom relief, with only sporadic nasal congestion elicited by cold stimulus.

### 2.3. Acupuncture method

**Figure 2.**
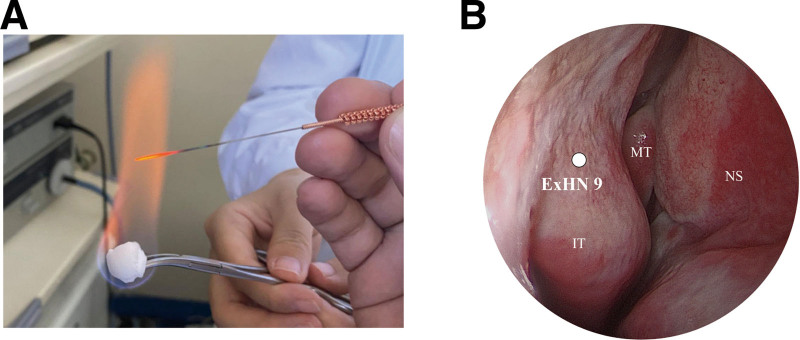
(A) The operation of fire needle: The front end of the hemostatic forceps clamped the alcohol cotton ball. After ignited the cotton, the proximal end of the Heshi fire needle was brought to a red-hot state via the alcohol flame. (B) Neiyingxiang (EX-HN 9) point: The acupoint were located in the lateral wall of the nasal cavity, the frontal attachment of inferior turbinate. The acupuncturist oblique insertion the acupoint toward the attachment of the middle turbinate at a depth of 0.3 to 0.5 cm, without needle retention. EX-HN 9 = Neiyingxiang, IT = inferior turbinate, MT = middle turbinate, NS = nasal septum.

The patient underwent a treatment regimen utilizing fire needles, administered by a physician specializing in acupuncture. The technique employed a specific needle—designated as a Heshi fire needle of medium to thick caliber, boasting a diameter of 0.8 mm. Hemostatic forceps were utilized to grasp an alcohol-soaked cotton ball. Subsequently, the cotton was ignited, and the proximal end of the fire needle was brought to a red-hot state via the alcohol flame (Fig. 2A). Following this, the needle was expeditiously inserted into the EX-HN 9 acupoint (Fig. 2B).

### 2.4. Outcome and follow-up

**Figure 3.**
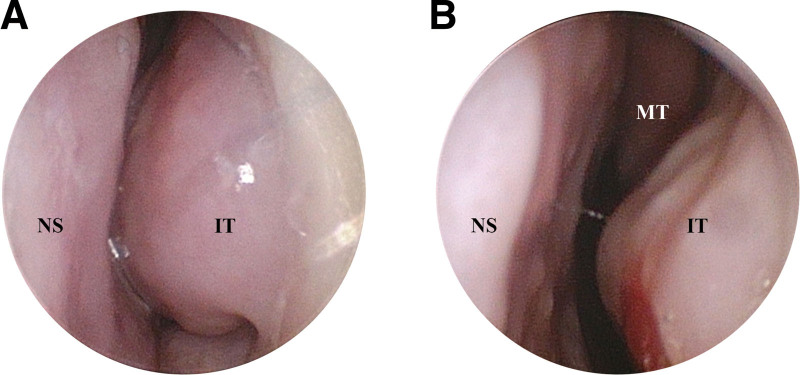
(A) The nasal endoscope of the left naris of the patient, before fire needle treatment. Nasal examination showed pale nasal mucosa with inferior turbinates swollen and edema, which could not see the middle turbinates. (B) The 10 min post-treatment nasal endoscope of the patient. Nasal examination showed a reduction in the swollen of inferior turbinates, which could see the middle turbinates. IT = inferior turbinate, MT = middle turbinate, NS = nasal septum.

**Figure 4.**
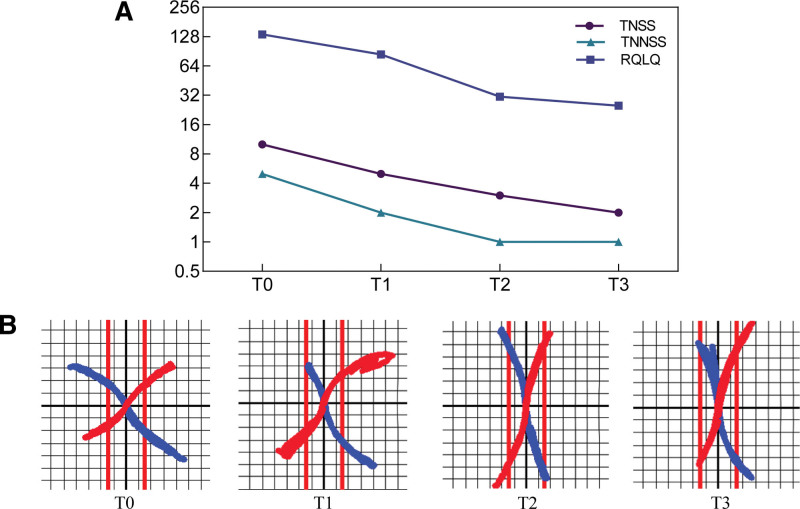
(A) The TNSS, TNNSS, and RQLQ scores of the patient. (B) The TNR of the patient. Red line represent right naris; Blue line represent left naris. The closer the line to the midline, the lower the TNR was. TNSS = total nasal symptoms score, TNNSS = total non nasal symptoms score, RQLQ = rhinoconjunctivitis quality of life questionnaire, TNR = total nasal resistance.

Prior to treatment, the patient manifested a markedly diminished quality of life due to chronic rhinitis, as evidenced by total nasal symptoms score, total non nasal symptoms score, and rhinoconjunctivitis quality of life questionnaire scores of 10, 5, and 135, respectively (T0). And The TNR was 0.41 kPa/(cm^3^·s). Nasal examination showed pale nasal mucosa with bilateral inferior turbinates swollen and edema, which could not see the middle turbinates. After fire needle treatment, the patient felt a significant improvement of nasal ventilation, and nasal examination showed a reduction in the swollen of inferior turbinates, which could see the middle turbinates (Fig. 3). Following 1 week of treatment, these scores were recalibrated to 5, 2, and 84, TNR was 0.25 kPa/(cm^3^·s), respectively (T1). At the 2-week (T2) and 4-week (T3) follow-up assessments, the scores further reduced to 3, 1, 31 and 2, 1, 25, TNR was 0.13, 0.16 kPa/(cm^3^·s) respectively (Fig. 4, Table 1). Post-treatment evaluations reveal a significant and enduring alleviation of the patient symptoms, with no reported recurrence within a 3-month observation period. And no negative side effects were noticed by the patients.

**Table 1:** Evaluation outcomes of fire needle acupuncture for the patient.

Date for record	TNSS	TNNSS	RQLQ	TNR (150 Pa)
	(0–12) scores	(0–7) scores	(0–168) scores	kPa/(cm^3^·s)
28-Oct-2022 (T0)	10	5	135	0.41
4-Nov-2022 (T1)	5	2	84	0.25
11-Nov-2022 (T2)	3	1	31	0.13
25-Nov-2022 (T3)	2	1	25	0.16

RQLQ = rhinoconjunctivitis quality of life questionnaire, TNNSS = total non nasal symptoms score, TNR = total nasal resistance, TNSS = total nasal symptoms score.

## 3. Discussion

PAR is characterized by an immunoglobulin E-mediated inflammation of nasal mucosa, it is a response to environmental aeroallergens that present throughout the year.^[6]^ The prevalence of PAR is increasing in recent years. For example, over the past 60 years, Switzerland has seen a threefold increase in the incidence of PAR and has caused a heavy socioeconomic impact.^[7]^ PAR has a negative impact on the patient quality of life, with poor mental and physical health, restricted social activities, and reduced ability to function effectively. PAR patient also suffer from sleep disturbance, leading to fatigue, irritability, memory deficits, daytime sleepiness, and depression.^[8]^ Current therapeutic methods for PAR management include allergen avoidance, allergen-specific immunotherapy, and pharma cotherapy with oral or intranasal second-generation antihistamines, intranasal glucocorticosteroids, and oral antileukotriene. Although these approaches can effectively control most PAR symptoms, they often present limitations such as incomplete relief, slow onset, <24-hour relief, and reduced efficacy with sustained use.^[9]^

Fire needle acupuncture therapy has been widely used in clinical treatment since ancient times in China. The concept of fire needle acupuncture finds its origins in the seminal work Huangdi Neijing. This technique involves the precise insertion of heated needles into specific acupoints within the human anatomy to ameliorate a variety of medical conditions. Fire needle acupuncture has the functions of activating the meridians, enhancing immunity, promoting blood circulation, inhibiting inflammation and preventing diseases.^[10]^ In modern practice, fire needle acupuncture has shown a good therapeutic effect, including taking less time, requiring fewer visits, producing effects more rapidly, and having fewer side effects compared with taking medicine.^[11]^ Contemporary research has corroborated the anti-inflammatory properties of fire needles and their efficacy in accelerating the recovery of inflamed tissues.^[12]^

Historically, some scholars have applied fire needles to manage allergic rhinitis, predominantly targeting the acupoints Yingxiang (LI 20), Shang Yingxiang (EX-HN 8), and Yintang (EX-HN 3), all of which are situated on the skin of craniofacial region.^[13]^ However, so many patients are unwilling to undergo this type of treatment because this methodology harbors a potential risk of cutaneous burns, which may cause disfigurement of the face. The EX-HN 9 is located in the lateral wall of the nasal cavity, the frontal attachment of inferior turbinate. It is an extra meridian point, which corresponds to the Yingxiang (LI 20) point of the large intestine meridian of hand yangming. This acupoint has demonstrated beneficial effects in clinical practice which including dredging meridians, activating collaterals, evacuating wind, and relieving stuffy nose. Some scholars find conventional acupuncture at EX-HN 9 has also yielded favorable outcomes in treating allergic rhinitis.^[14]^ Nonetheless, traditional acupuncture necessitates frequent, short-interval sessions (every 2 to 3 days) with prolonged needle retention times, typically spanning 20 to 30 minutes. Many patients are unable to complete the full course of treatment. Given that the EX-HN 9 acupoint is situated within the nasal cavity, elongated needle retention could also exacerbate patient discomfort.

The mucosa of nasal at EX-HN 9 point is innervated by 3 nerves, including sensory nerve (nasociliary nerve), parasympathetic nerve (great superficial petrosal nerve) and sympathetic nerve (deep petrosal nerve).^[15]^ Acupuncture could stimulate the nasal nerve, activating the local blood and lymphatic circulation of the nose, shrinking the blood vessels around the inferior turbinate, reducing the sensitivity of the nasal mucosa to relieve the enlargement of the inferior turbinate and improve the ventilation of the nose.^[16]^ In an innovative departure from conventional methods, we employed fire needle acupuncture targeting the EX-HN 9 acupoint, resulting in notable therapeutic benefits. Additional studies have indicated that laser irradiation, radiofrequency, and chemical cauterization of the turbinate mucosa can attenuate vascular permeability and glandular secretion through the inhibition of local nerve sensitivity.^[17,18]^ Correspondingly, fire needle methodology achieves similar outcomes, offering ease of application through anterior rhinoscopy, obviating the necessity for an operating room or endoscopic intervention.

## 4. Conclusion

In summary, the application of fire needle acupuncture in the treatment of allergic rhinitis yielded highly efficacious outcomes, characterized by sustained symptom relief extending over a 3-month period. Future research endeavors should focus on expanding the sample size to further validate the observed results, as well as extending the follow-up period to assess the longevity of treatment efficacy.

## Acknowledgments

The authors are grateful to the patient for participation in the study and allowing for publication of this case report.

## Author contributions

**Conceptualization:** Suying Guo, Jianwei Ai.

**Methodology:** Yaqin Wang, Yuezhi Kang, Shaoting Huang.

**Supervision:** Man Wang, Jingyi Zhao.

**Writing – original draft:** Suying Guo.

**Writing – review & editing:** Jianwei Ai, Junge Wang.
